# Correlation between positron emission tomography and Cerenkov luminescence imaging *in vivo* and *ex vivo* using ^64^Cu-labeled antibodies in a neuroblastoma mouse model

**DOI:** 10.18632/oncotarget.11795

**Published:** 2016-09-01

**Authors:** Florian C. Maier, Julia Schmitt, Andreas Maurer, Walter Ehrlichmann, Gerald Reischl, Konstantin Nikolaou, Rupert Handgretinger, Bernd J. Pichler, Wolfgang M. Thaiss

**Affiliations:** ^1^ Werner Siemens Imaging Center, Department of Preclinical Imaging and Radiopharmacy, Eberhard Karls University Tübingen, Tübingen, Germany; ^2^ Department of Radiology, Eberhard Karls University Tübingen, Tübingen, Germany; ^3^ University Childrens Hospital, Eberhard Karls University Tübingen, Tübingen, Germany

**Keywords:** Cerenkov luminescence imaging, positron emission tomography, neuroendocrine tumor, mouse imaging, monoclonal antibody

## Abstract

Antibody-based therapies gain momentum in clinical therapy, thus the need for accurate imaging modalities with respect to target identification and therapy monitoring are of increasing relevance. Cerenkov luminescence imaging (CLI) are a novel method detecting charged particles emitted during radioactive decay with optical imaging. Here, we compare Position Emission Tomography (PET) with CLI in a multimodal imaging study aiming at the fast and efficient screening of monoclonal antibodies (mAb) designated for targeting of the neuroblastoma-characteristic epitope disialoganglioside GD2. Neuroblastoma-bearing SHO mice were injected with a ^64^Cu-labeled GD2-specific mAb. The tumor uptake was imaged 3 h, 24 h and 48 h after tracer injection with both, PET and CLI, and was compared to the accumulation in GD2-negative control tumors (human embryonic kidney, HEK-293). In addition to an *in vivo* PET/CLI-correlation over time, we also demonstrate linear correlations of CLI- and γ-counter-based biodistribution analysis. CLI with its comparably short acquisition time can thus be used as an attractive one-stop-shop modality for the longitudinal monitoring of antibody-based tumor targeting and *ex vivo* biodistribution.

These findings suggest CLI as a reliable alternative for PET and biodistribution studies with respect to fast and high-throughput screenings in subcutaneous tumors traced with radiolabeled antibodies. However, in contrast to PET, CLI is not limited to positron-emitting isotopes and can therefore also be used for the visualization of mAb labeled with therapeutic isotopes like electron emitters.

## INTRODUCTION

Non-invasive visualization of tumor occurrence and therapeutic monitoring with a special focus on early therapeutic response assessment are increasingly gaining importance in clinical routine. Especially with the availability of new therapeutic agents such as highly specific antibodies targeting tumor or vascular epitopes, the non-invasive detection of such expressed epitopes *in vivo* is gaining attention. Additionally, conventional tumor diameter based imaging strategies often show limited accuracy in terms of therapy response evaluation (e.g. a novel therapeutic approach might show no significant change or even an increase in tumor size when an anatomy-based imaging readout is used [1]). Furthermore, the rapid change of the expression of a therapeutic target under therapy demands fast assessment of the tumor phenotype and the efficacy of a given molecular treatment. Thus, the non-invasive identification of tumor specific epitopes and possible changes of their expression under therapy are important clinical imperatives and would be of high predictive value in considering potential therapy response [2].

Epitope specific antibodies can be used to detect target molecules and to evaluate the accessibility of these structures, e.g. in metastases. By administration of tracers at picomolar concentration, Positron Emission Tomography (PET) is able to detect metabolically active sites in healthy and diseased tissue. The identification of potential therapeutic targets, as well as the *in vivo* evaluation and stratification of molecular therapeutics while avoiding pharmacodynamic effects are clear advantages of PET [3, 4]. Thus, combining the exceptional detection sensitivity of PET with the outstanding selectivity of specific, radiolabeled antibodies makes it feasible to study epitope expression patterns in oncological studies in laboratory animals. However, PET imaging requires expensive tomographic systems and is usually characterized by measurement times ranging from 10-20 min for static imaging studies and up to 60-90 min for dynamic PET assessments [5, 6].

Apart from a variety of studies that used PET for the preclinical evaluation of antibody-coupled tracers, Cerenkov Luminescence Imaging (CLI) is gaining interest as a novel method for the detection and evaluation of radiolabeled molecules in preclinical models [7–10]. CLI enables the detection of radioactive decays (β^+^ and β^−^, theoretically also α) with an optical imaging (OI) system via the phenomenon of visual light emission that is indirectly induced by charged particles. Those particles such as positrons emitted from unstable nuclei used for PET imaging polarize the surrounding dipolar molecules if traveling faster than the speed of light in the respective medium. While these molecules return to their equilibrium state, Cerenkov radiation is emitted, consisting of photons with a continuous spectrum at a wavelength depending on the charged particle energy that is being emitted. A maximum is emitted in the ultraviolet/blue range of the light spectrum, however, ranges up to more than 800 nm [9, 11]. Sensitive CCD cameras, as present in state-of-the-art OI-devices, can detect these photons – typically in the range from 500-800 nm. As state-of-the-art OI-systems are relatively cheap in comparison to PET-systems, widely available throughout small-animal research institutes worldwide, and as typical OI-studies only require acquisition times in the sub-second to second range, CLI is becoming increasingly interesting for fast and efficient high-throughput studies. The theoretical background of CLI and current applications have recently been reviewed [12]. Additionally, the feasibility of CLI in humans has also recently been demonstrated [13, 14], providing CLI with an important translational aspect.

The tumor-specific epitope disialoganglioside GD2 can be found as surface marker on a variety of neuroendocrine tumors such as neuroblastoma [15–17]. As neuroblastomas represent a highly aggressive tumor entity that is difficult to assess by means of non-invasive imaging, we aimed to screen GD2-targeted monoclonal antibodies for target specificity. The basic characterization of specific antibody libraries is possible with PET; however, the use of a high-throughput modality like CLI enables timesaving screening assays both *in vivo* and *in vitro* [18].

Thus, in this study, we used a subcutaneous GD2-expressing neuroblastoma mouse model along with a control tumor and traced the lesions with ^64^Cu-DOTA-labeled antibodies longitudinally with PET and CLI *in vivo*, while magnetic resonance imaging (MRI) was used to determine tumor size and for anatomical guidance during PET data-evaluation. Furthermore, we aimed to investigate CLI as a method beyond detecting radioactive decay but to also assess the *ex vivo* organ biodistribution in comparison to established γ-counter biodistribution.

## RESULTS

### CLI phantom study of ^64^Cu decay

**Figure 1:**
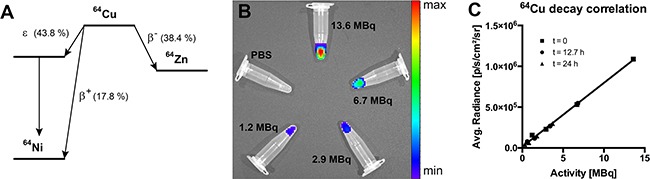
CLI phantom study **A.** Decay scheme for ^64^Cu with probabilities for β^+^, β- and ε decay (taken from [19]). **B.** CLI phantom study setup (activities: 13.6 MBq, 6.7 MBq, 2.9 MBq and 1.2 MBq). **C.** Correlation of CLI-measured and theoretically calculated decay of ^64^Cu-filled phantoms for time points t = 0, t = 12.7 h and t = 24 h (R^2^ = 0.997, p < 0.01); data are displayed as the mean.

First, the feasibility for stable and reliable detection of Cerenkov radiation from ^64^Cu-solutions was demonstrated. As ^64^Cu decays by β^+^, β^−^, ε and auger electron emission, robust CLI signals were expected (the decay mode of ^64^Cu is delineated in Figure 1A). In the case of ^64^Cu, Cerenkov luminescence is released from high-energy emission positrons (maximum positron energy: 653.03 keV, mean positron energy: 278.21 keV, [19] above the calculated threshold for Cerenkov luminescence emission of 263 keV [20] at an estimated refractive index of 1.4 [21] for mouse tissue. Cerenkov luminescence from ^64^Cu at different activities, down to typical *in vivo* activity concentrations were used for the phantom study (13.6 MBq, 6.7 MBq, 2.9 MBq and 1.2 MBq, Figure 1B). The radioactive decay was measured via CLI for 24 h with initially measured average radiances of 1.09, 0.53, 0.23, and 0.16 × 10^6^p/s/cm^2^/sr, respectively (Figure 1C), average radiance and activity concentration were linearly correlated over the course of the 24 h measurement (R^2^ = 0.997, Figure 1C). Further, the measured decay was in line with theoretically calculated values, mono-exponential fitting ($y=y_{0}\cdot e^{-\tau_{i}t}$) resulted in an R^2^ of 0.99 and decay constants *τ_i_* of 1.512*10^−5^ s^−1^, 1.511*10^−5^ s^−1^, 1.502*10^−5^ s^−1^ and 1.516*10^−5^ s^−1^ for the 13.6 MBq, 6.7 MBq, 2.9 MBq and 1.2 MBq activity concentrations, respectively (Supplementary Figure S1). Differences in the assessed decay constants are most likely attributable to differential signal-to-noise ratios; however, the mean fitted decay constant 1.510*10^−5^ ± 5.909*10^−8^ s^−1^ matched the calculated ^64^Cu-decay constant of 1.516*10^−5^ s^−1^; ^64^Cu-decay constant was calculated from the literature half-life value of ^64^Cu of 12.7 h [19].

### *In vivo* PET, MRI and CLI studies

**Figure 2:**
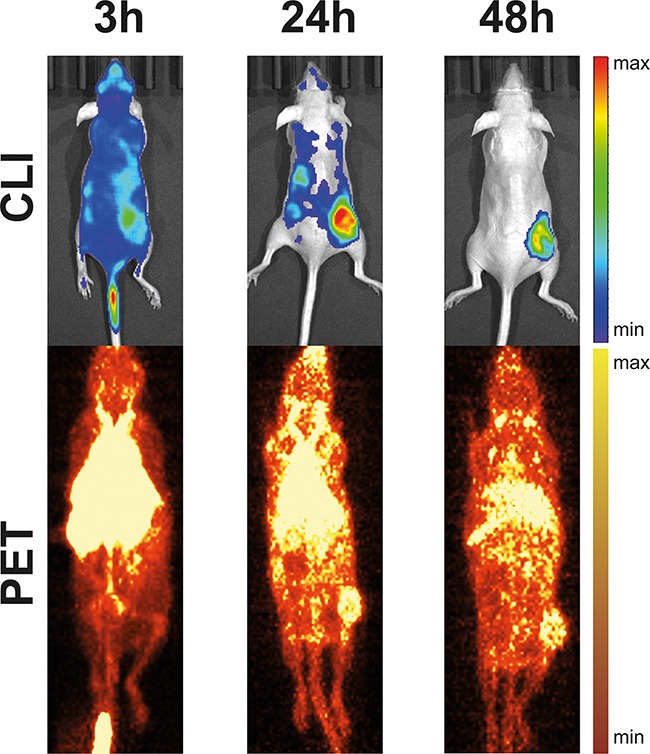
Example for CLI- and PET-acquisitions from a LS-bearing SHO-mouse injected with ^64^Cu-DOTA-ch14.18 CLI-acquisitions (top, 1 min acquisition time) and maximum intensity projections of static PET-scans (bottom, 10 min acquisition time) 3 h, 24 h, and 48 h after injection of ^64^Cu-DOTA-ch14.18 show good visual agreement for tumor localization and uptake. Notably, CLI allowed earlier identification of ^64^Cu-DOTA-ch14.18-uptake in subcutaneous neuroblastoma at the right hind-leg.

After ensuring reliable CLI-measurements in phantoms, we next aimed to assess the *in vivo* correlation between PET and CLI. Therefore, we targeted the disialoganglioside GD2 with a specific ^64^Cu-labeled monoclonal antibody (mouse-human chimeric ch14.18). The antibodies were injected intravenously (i.v.) into severe immune deficient hairless outbred (SHO) mice bearing subcutaneous neuroblastomas (GD2 positive cell line [22], in the following termed LS) or control tumors (human embryonic kidney cells, HEK-293, GD2 negative) according to the following groups (Table 1). A representative example for ensuing PET- and CLI-measurements taken 3 h, 24 h and 48 h post injection (p.i.) is displayed in Figure 2 (all measurements show the same LS-bearing SHO-mouse injected with ^64^Cu-DOTA-ch14.18, Table 1). Qualitatively, CLI enabled tumor-detection *via*^64^Cu-DOTA-labeled antibodies at all time-points, especially already at 3 h p.i., whereas the first tumor-identification based on PET was feasible 24 h p.i.

**Table 1:** MRI-derived tumor volumes assessed for all experimental groups

Tumor-type	^64^Cu-DOTA-mAb	MRI-derived tumor volume
**1. LS**	ch14.18 (n = 5)	94.1 ± 31.7 mm^3^
**2. HEK-293**	ch14.18 (n = 5)	99.8 ± 58.6 mm^3^

No significant size differences were observed between the groups (unpaired t-test, p > 0.85). LS: GD2 expressing neuroblastoma cells; HEK-293: human embryonic kidney cells. Data are displayed as mean ± SD.

**Figure 3:**
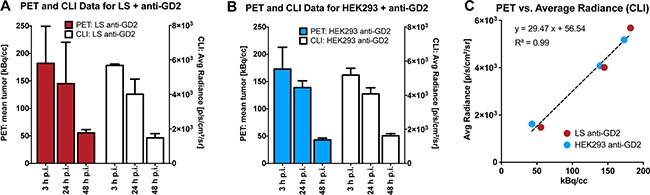
Quantification and correlation of PET- and CLI-assessed mAb-uptake in LS- and HEK293-tumors Quantitative PET- (color bars, **A,B**) and CLI-data (open bars, **A,B**) for experimentally investigated mouse groups; **A.** LS + anti-GD2, **B.** HEK293 + anti-GD2. PET- and CLI-acquisitions yielded comparable mAb-uptake dynamics in experimental groups. Moreover, PET and CLI quantitation displayed a linear correlation, independent of the study group (**C.**, R^2^ = 0.99, p<0.0001). Data are displayed as the mean ± SD (**A,B**) or as the mean (**C**).

Quantification of PET and CLI revealed that LS-bearing SHO-mice injected with ^64^Cu-DOTA-ch14.18 (n = 5) displayed a tumor-uptake of 182 ± 76 kBq/cc at 3 h p.i., 145 ± 70 kBq/cc at 24 h p.i. and of 55 ± 6 kBq/cc at 48 h p.i. (Figure 3A, PET, Table 1). As described in Materials and Methods, CLI was performed directly after the PET-measurements. Corresponding CLI-data (Figure 3A, CLI) were assessed to 5.78 ± 0.08 (3 h p.i.), to 4.01 ± 0.91 (24 h p.i.) and to 1.48 ± 0.22 × 10^3^p/s/cm^2^/sr (48 h p.i), respectively.

In a second group of SHO-mice, we aimed to investigate the uptake of the GD2-specific mAb ch14.18 in a control tumor line devoid of the target protein expression. Thus, HEK-293-bearing SHO-mice (n = 5) were measured with ^64^Cu-DOTA-ch14.18 employing PET and subsequent CLI. HEK-293 tumors showed a PET activity of 173 ± 40 kBq/cc (3 h p.i.), 139 ± 12 kBq/cc (24 h p.i.) and 43 ± 3 kBq/cc (48 h p.i.) after injection of ^64^Cu-DOTA-ch14.18 (Figure 3B, PET) and corresponding CLI data of 5.18 ± 0.01 (3 h p.i.), 4.08 ± 0.35 (24 h p.i.) and 1.62 ± 0.12 × 10^3^p/s/cm^2^/sr (48 h p.i.; Figure 3B, CLI), respectively.

To obtain a quantitative assertion of CLI using PET as a gold-standard, we correlated *in vivo* CLI and PET data (Figure 3C), and detected a linear correlation between PET and CLI tumor uptake with an R^2^ of 0.98 (r = 0.99) for the LS-^64^Cu-DOTA-ch14.18 group and an R^2^ of 0.99 (r = 0.99) for the HEK-293-^64^Cu-DOTA-ch14.18 group. Pooled data for all animals displayed a comparable PET and CLI correlation (R^2^ = 0.99, r = 0.99, Figure 3C). Importantly, no statistically significant differences in MRI-derived tumor-size were detected among all measured animal groups, so tumor-size effects potentially influencing both CLI- and PET-data quantification could be excluded (unpaired t-test; p > 0.85; Table 1).

### *Ex vivo* biodistribution with CLI

**Figure 4:**
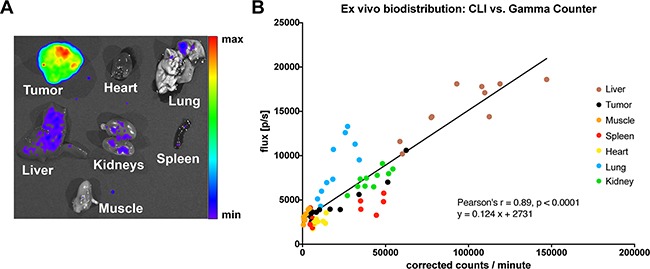
Ex vivo biodistribution **A.** Example of an *ex vivo* CLI-biodistribution, in this case from a LS-bearing SHO-mouse injected with ^64^Cu-DOTA-ch14.18 (biodistribution was performed 48 h after mAb-injection). **B.** CLI- and γ-counting-based *ex vivo* analysis displayed a significant linear correlation (n = 10 mice, n = 70 organs; R^2^ = 0.79, p < 0.0001). Notably, *ex vivo* liver CLI-signals are higher in comparison to tumor-signals, opposing the *in vivo* situation, where liver signals are quenched in planar CLI-acquisitions (Figure 2), the observed discrepancies depending on the measured organ-type might be related to tissue density and thus, differential tissue quenching.

After the last dedicated PET and MRI measurement, we analyzed the organ biodistribution from all animals *ex vivo* with CLI (acquisition time 3 min). Organs were subsequently transferred to γ-counter tubes and inserted in a Wallac 1480 WIZARD γ-counter for correlative measurements of ^64^Cu-DOTA-mAb uptake, the gold standard for biodistribution studies in nuclear imaging. An example of a CLI-based biodistribution measurement is given in Figure 4A. Correlation analysis (*ex vivo* CLI and γ-counting) was performed by pooling all investigated organs (tumor, heart, lungs, liver, spleen, kidneys, and muscle, n = 70 organs) yielding a significant linear correlation (Pearson's r = 0.89, p < 0.0001 Figure 4B).

## DISCUSSION

The first application of Cerenkov radiation for the use in preclinical and eventually clinical applications in 2009 [7] led to the rapid realization that radioactive tracers can be easily detected with state-of-the-art OI-devices. Several studies already pointed towards the feasibility and benefits in using Cerenkov radiation as a method to detect radioactive agents with optical imaging, ranging from basic phantom feasibility studies to *in vivo* applications for, e.g., ^18^F-FDG detection in brown adipose tissue [23] where the benefits of the superficial position and fast acquisition times were demonstrated. The authors could show that two-minute acquisitions were suitable to monitor the effect of different anesthetics on activation of brown adipose tissue. Further studies delineate benefits of CLI where PET is not suitable. Lohrmann and colleagues demonstrated radiation dose calculation with ^90^Y, which is limited with PET due to only rare internal pair production [24]. A novel application has recently been demonstrated with endoscopic CLI-detection in animal and human studies, providing novel detection methods and thus broadening the application fields for radioactive tracers [24, 25]. Here, we tested the efficacy of a ^64^Cu-DOTA-labeled monoclonal antibody to trace the expression of the specific tumor epitope GD2 in a mouse model of subcutaneous neuroblastoma (in addition to a control tumor line devoid of GD2) over the time course of 48 hours. The state-of-the-art *in vivo* tumor-uptake characterization via a combination of dedicated PET and MRI (along with standard *ex vivo* γ-counting) in two different xenografts was directly compared to CLI, to yield a predication on the value of CLI-measurements in antibody based high-throughput imaging studies aiming at the identification of novel, promising imaging agents. Even though only data-analysis for two-dimensional, planar datasets can be performed for CLI compared to a three-dimensional full-volume-analysis (in the study at hand) determined by T_2_-weighted MR imaging and dedicated PET, combining morphological and functional information, the *in vivo* CLI experiments demonstrate an excellent correlation between tumoral CLI- and PET-quantifications across study groups and time points (3 h, 24 h and 48 h p.i.). Thus, it can be argued that CLI is a reliable method to monitor the targeting of superficial subcutaneous tumors with ^64^Cu-labeled antibodies – enabling a rapid screening of novel, potential candidate-antibodies for targeted imaging and radiotherapy.

Compared to a standard small animal PET, the CLI acquisition can be achieved faster (one minute compared to ten minutes). The increased acquisition speed holds also true for the CLI-biodistribution that was achieved faster than γ-counter based biodistribution (45 minutes with a 3 min acquisition for 7 organs per mouse in CLI vs. 70 minutes for 70 organs with one minute γ-counter acquisition). CLI has been used for organ biodistribution studies and for *ex vivo* correlative analysis previously [26, 27]. In line with previous publications, we found a linear correlation between CLI and γ-counter biodistribution-assessments, if pooling all organs (r = 0.89, p<0.0001). However, no previous publication made use of as many different organs as described in our study, and thus, it was not reported previously that there can be a quite substantial deviation in the CLI-γ-counting correlative analysis – depending on the examined organ. For all organs, the correlation was found to be linear between CLI and γ-counting, however, with differing slopes. These organ-specific deviations in the linear relationship between CLI and γ-counting might be related to different tissue densities and to the amount of the major quenching factor in tissue in optical imaging studies, the tissue fractional blood volume (e.g. if lung and spleen are compared to each other, Figure 4B). The basic question about the comparability of a biodistribution-quantification that is based on CLI-measurements versus γ-counter-based data remains unanswered, as CLI can only be normalized based on the bright field image, while γ-counter data is usually normalized per gram wet tissue, and, as stated above, CLI signals most likely depend on tissue density and fractional blood volume. In the study at hand, we used the total CLI-flux and the total detected γ-counts for our correlative analysis. Thus, CLI-based biodistributions are feasible for rapid screenings which can be used for both fast assessments, and, in the absence of a γ-counter, for *ex vivo* biodistribution control. In comparison to a PET-γ-counter setup, a CLI-setup only requires one instrument – reducing acquisition costs dramatically. Thus, CLI-setups might be useful for laboratory start-ups.

The substantial unspecific liver-signals typically observed in ^64^Cu-based PET-imaging studies (e.g., caused by trans-chelation to superoxide dismutase in the liver [28]) are suppressed in CLI by quenching of adjacent overlying tissue. This otherwise undesired effect increases the contrast to noise ratio of the specific CLI signals and simplifies the identification of antibody-labeled tumor tissue in this case. In line with this, CLI enabled the visualization of antibody-targeting as early as 3 h p.i., while PET-based tumor identification was possible 24 h p.i. The chosen tumor inoculation site thus appears to be suitable for robust CLI-studies with ^64^Cu-labeled antibodies or peptides. Further, as the excretion route *via* the liver is under regular conditions, a commonly shared principle of radiometals, this should be also true for other metals, e.g. ^68^Ga [29]. Limitations of CLI-based imaging studies are the limited penetration depth and the high amount of diffusely scattered photons in the mouse tissue – owing to the high amount of emitted photons in the blue range of the visible light spectrum that is detectable with state-of-the-art OI-systems. Moreover, due to the already mentioned shortcomings, CLI-data are not as easily amenable to kinetic modeling of acquired data as PET-data are.

Recent studies demonstrate that antibody-based tracer visualization with CLI can be further optimized using quantum-dots or other fluorophores excited by Cerenkov luminescence [10, 30], especially in terms of enhancing the signal-to-noise ratio. However, this requires special preparations and potentially longer acquisition times.

Although striking, the comparable antibody uptake of the investigated tumor lines was previously investigated and will be discussed in-depth elsewhere. We provide a short summary of relevant results in the Supplementary Data. However, it should be pointed out that the goal of this study was the evaluation of CLI as a novel specificity screening method for radiolabeled antibodies. While non-superior to the individual standards for the evaluation of *in vivo* and *ex vivo* tracer distribution analysis, CLI with the need for only one imaging device showed reasonable correlations for both, *in vivo* imaging of subcutaneous tumors and *ex vivo* biodistribution. Therefore, we propose CLI as a one-stop-shop modality that allows quick assessment and screening of radiolabeled antibodies. From this body of data, it can be concluded that CLI can be used instead of PET for fast and efficient screening of antibody-based biomarker libraries, aiming at specific tumor-localization – although limited to superficial detection.

## MATERIALS AND METHODS

### Animals

All animal experiments were performed according to current national and international guidelines after permission was granted by local authorities (Regierungspräsidium Tübingen). Animals were kept under standardized conditions (20 ± 1°C room temperature, 50 ± 10 % relative humidity, and 12 h light-dark cycle). 6-week-old severe immune deficient hairless outbred (SHO) mice (Charles River Laboratories, Sulzfeld, Germany) were injected subcutaneously at the right lower flank with 6 × 10^6^ GD2 expressing neuroblastoma cells (LS, n = 5) or human embryonic kidney cells (HEK-293, control, n = 5) in PBS with 50 % Matrigel (BD Biosciences, Franklin Lakes, NJ, USA) under a 1.5 % isoflurane anesthesia (CP-Pharma Handelsgesellschaft mbH, Burgdorf, Germany).

### Cells

LS-cells (human GD2-positive neuroblastoma cell line [22, 31]) were purchased from DSMZ (Braunschweig, Germany, ACC-675), and HEK293 cells were obtained from ATCC (ATCC CRL-1573, Manassas, Virginia, USA). Cells were cultured in RPMI 1640 medium, with 100 U/mL penicillin, 100 mg/L streptomycin and 10 % fetal calf serum (FCS) (all from Biochrom GmbH, Berlin, Germany) at 37°C in a humidified atmosphere of 5 % CO_2_ in a cell culture cabinet (HeraSafe KS18, Thermo/Kendro, Dreieich/Hanau, Germany). GD2-expression of LS-cells was confirmed with FACS-analysis.

### Antibodies

The mouse-human chimeric IgG1,κ antibody ch14.18 (specific for the disialoganglioside GD2 [32]) was manufactured by POLYMUN GmbH (Vienna, Austria) and used for all experiments.

For PET imaging the antibody was conjugated with 2,2′,2”,2′”-(1,4,7,10-tetraazacyclododecane-1,4,7,10-tetrayl)tetraacetic acid (DOTA) *via* the respective *N*-hydroxysuccinimide (NHS) ester and radiolabeled with ^64^Cu^2+^ as described in [33]. Briefly, the antibody was adjusted to pH 7.5 by ultrafiltration (Amicon Ultra-15, MWCO 40 kDa, Merck Millipore) with 0.1 M sodium phosphate (treated with Chelex 100, Na^+^ form, Sigma-Aldrich). Then, a 55fold molar excess of DOTA-NHS (Chematech, Dijon, France) was added and incubated at 4°C for 24 hours. Excess of chelator was removed by ultrafiltration with chelex-treated 0.25 M ammonium acetate (pH 7.0).

^64^Cu was produced by the ^64^Ni(p,n)^64^Cu reaction with 12.4 MeV protons from a PETtrace 860 cyclotron (GE Healthcare, Uppsala, Sweden). ^64^Cu was isolated by ion exchange chromatography using a modified procedure according to McCarthy et al. [34] and redissolved in 0.1 M HCl.

For radiolabeling of the antibody, the ^64^CuCl_2_ solution was buffered with 1.5 volumes of 10x PBS (Carl Roth, Karlsruhe, Germany) and the antibody (1 μg/MBq) was added and incubated for 60 minutes at 42°C. Thin layer chromatography (Polygram SIL G/UV254 nm, Macherey-Nagel, Dueren, Germany, 0.1 M sodium citrate pH 5) and analysis on a Cyclone Plus phosphorimager (Perkin Elmer, Waltham, Massachusetts, USA) showed complete (radiochemical purity of 94.6 %) incorporation of ^64^Cu.

### CLI-phantom study

13.6 MBq, 6.7 MBq, 2.9 MBq and 1.2 MBq of ^64^Cu in 100 μL were prepared in 1.5 mL Eppendorf cups. CLI-acquisitions were performed with an IVIS Spectrum optical imaging system (Perkin Elmer, Wellesley, MA, USA) with the following settings: open filter acquisition, binning 16, f-stop 1, acquisition time 1 min. Bright field images were acquired with: binning 2, f-stop 8, acquisition time 0.2 s. Images were taken once every hour for 24 h.

### *In vivo* PET, MRI and CLI studies

28 (LS) and 21 (HEK293) days after tumor inoculation, anesthetized mice (1.5 % isoflurane) were intravenously injected with 10.4 ± 0.9 MBq anti-GD2 antibodies in a formulation of 50 μg net antibody in 50 μL of sterile saline. Static PET acquisitions (10 min) were performed 3 h, 24 h, and 48 h after tracer injection using a dedicated micro-PET scanner (DPET, Siemens Preclinical Solutions, Knoxville, USA) with a spatial resolution of 1.4 mm full width at half maximum in the center-field-of-view (center-FOV) and an axial FOV of 12.7 cm [35]. All PET-scans were acquired with an energy window of 350 – 650 keV and a coincidence timing window of 3.432 ns. PET scans were normalized. No attenuation correction was applied, as only mice on thin plastic beds were scanned; thus, we did not expect anisotropic attenuation. PET-images were reconstructed employing two-dimensional *ordered-subset-expectation-maximization* (OSEM2D), an image zoom of 1 and a 128 × 128 matrix, resulting in a final resolution of 0.79 × 0.79 × 0.80 mm^3^.

Subsequent on-bed transfer of the animals to a 7 Tesla small animal MRI scanner (Clinscan, Bruker Biospin, Ettlingen, Germany) ensured anatomical MRI scans at identical bed positions. T_2_-weighted anatomical MR-images were acquired using the following 3D-spoiled turbo spin echo sequence: matrix size: 256 × 161, FOV: 35 × 57 mm^2^, repetition time (TR) = 3000 ms, echo time (TE) = 205 ms, slice thickness (ST) = 0.22 mm.

Animal transfer to the OI device followed again without intermediate awakening of the animals; however, this step required removing the animals from the PET and MRI compatible beds. While acquiring CLI, the body temperature was maintained with the installed heating plate at 37°C. Acquisitions (open filter, binning 16, f-stop 1, acquisition time 1 min) were performed at a 12.5 cm FOV. Bright field images were acquired with binning 2, f-stop 8 and 0.2 s acquisition time. Further, all CLI-data were normalized (correction for cosmic rays and flat field). CLI-images are shown in all figures with software binning 8 and smoothing 5, while PET-images are shown as maximum intensity projections. Values were expressed as either photons / second (p/s) or average radiances (p/s/cm^2^/steradian).

### *Ex vivo* CLI and γ-counting studies

After the last imaging time-point (48 h after antibody injection), mice were sacrificed by cervical dislocation under deep isoflurane anesthesia; tumor, heart, lungs, liver, spleen, kidneys and gastrocnemius muscle were prepared (n = 70 organs from n = 10 animals). CLI-acquisitions of the explanted organs followed immediately after preparation. Acquisitions were conducted with open filter, binning 16, f-stop 1 for three minutes. Furthermore, all removed organs were measured along with aliquoted ^64^Cu activity solutions in quadruplicate in a Wallac 1480 WIZARD γ-counter (Perkin Elmer, Wellesley, MA, USA) using an energy window of 350 – 650 keV, to mimic the PET-measurement energy window. Decay corrected radioactivity was normalized according to wet sample weight and results were expressed as counts/min or percent injected dose per gram wet tissue (%ID/g).

### PET and CLI data analysis

For all data analysis of CLI- and PET-data, raw data were used. Data analysis was conducted with Inveon Research Workplace (IRW, Siemens Preclinical Solutions, Knoxville, USA). PET and MRI data were co-registered for exact, MRI-guided delineation of volumes of interest (VOI). PET-VOIs were drawn manually for tumors. Living Image 4.4 (Perkin Elmer, Wellesley, MA, USA) was used for CLI data analysis. CLI-Regions of interest (ROI) were drawn manually for *in vivo* tumors and *ex vivo* organs using the individually acquired bright-field images, standardized background ROIs were used for background subtraction. Neither PET-, nor CLI-data were decay corrected. As mentioned before, obtained CLI-values were either expressed as photons / second (p/s) or average radiances (p/s/cm^2^/steradian), while PET-data were either expressed as kBq / cm^3^ (kBq/cc) or as percent of the injected dose / cm^3^ (%ID/cc).

### Statistics

Statistical testing and linear fitting analysis were conducted with Prism 5 (GraphPad software, La Jolla, CA, USA). Mono-exponential fitting was performed with the Origin 8.0 Pro software package (OriginLab, Northhampton, USA). Before statistical tests were applied, it was ensured that all general requirements like data normality or homoscedasticity for statistical tests were met. All data are reported as arithmetic mean ± one standard deviation (SD). Pearson's correlation coefficient r is used to report correlation coefficients between PET and CLI data.

## SUPPLEMENTARY MATERIAL FIGURES


