# Supplementary material for: Correlation between positron emission tomography and Cerenkov luminescence imaging in vivo and ex vivo using 64Cu-labeled antibodies in a neuroblastoma mouse model
Source: Oncotarget. 2016 Sep 1;7(41):67403–11. doi: 10.18632/oncotarget.11795 (PMC5341884; doi:10.18632/oncotarget.11795)
Supplement: Supplementary file 1 [file oncotarget-07-67403-s001.pdf]

# Correlation between positron emission tomography and cerenkov luminescence imaging *in vivo* and *ex vivo* using $^{64}$Cu-labeled antibodies in a neuroblastoma mouse model

## SUPPLEMENTARY DATA

### $^{64}$Cu-DOTA-ch14.18 shows comparable uptake in GD2-positive LS- and in GD2-negative HEK293 tumors

Strikingly, we found no significant difference in tumor uptake of $^{64}$Cu-DOTA-ch14.18 in GD2-positive LS- and in GD2-negative HEK293 tumors. As the GD2-binding specificity of ch14.18 has been proven in the past, we assessed deviations of the immunoreactive fraction as potential source for this phenomenon; the detailed results will be published in an upcoming publication focusing on GD2 antibodies and GD2-PET imaging. Briefly, chelator-conjugation and subsequent $^{64}$Cu-labeling of the used GD2-specific mAb resulted in a dramatically reduced immunoreactive fraction (reduction to 6.4 %), albeit the afore via FACS, ELISA and IHC assessed GD2-specificity of the used mAb, ch14.18. Potential reasons for this dramatic reduction in immunoreactive fraction could be the presence of a crucial lysine residue in the specificity domain of ch14.18, which could be blocked by coupled chelator, and radiolysis of the mAb caused by $^{64}$Cu.

Most important, the aim of our paper was the comparison of CLI and PET quantification. The investigated antibodies and cell lines were used to determine the correlation of CLI and PET quantification in one biological model system.

### Advantages and limitations of CLI

Imaging brain tumors, brain metastasis or tumors deeply-located in healthy tissue with CLI is much less favorable in comparison to a subcutaneous xenograph model – as the neuroblastoma model used in this study. Attenuation and scattering by dense tissue like bone or by the vast tissue mass surrounding deeply-located lesions also causing high rates of scatter, attenuation and light diffusion are major limitations of CLI in comparison to PET. However, for fast and efficient specificity-screening of antibodies targeted at tumor-type characteristic epitopes, we suggest using CLI and subcutaneous xenograph models, with all limitations and benefits of this kind of tumor mouse model system and imaging modality. An additional limitation of CLI is also shown in our study, as *in vivo* whole-body biodistribution of signal intensity is clearly different between PET and CLI. As the signal intensity of PET can be considered as ground-truth in this comparative imaging setup, the altered signal intensity in CLI-images is most likely caused by tissue attenuation and scatter, and by the low tissue-penetration of light in the blue range of visible light. Only relying on *in vivo* CLI-imaging might thus result in an incorrect data interpretation regarding tracer biodistribution – *ex vivo* control experiments by both *ex vivo*-CLI of excised organs and additional $\gamma$-counting are thus crucial for correct conclusions about tracer-specificity, total uptake in tissue of interest or tracer-biodistribution, as shown in this study.

The demonstration of this principle is the major aim of this manuscript, imaging of further tumor-model systems is beyond the scope of this report. Spinelli et al. demonstrate in a recent study the merit of CLI during neurosurgery using $^{90}$Y-DOTATOC as CLI-tracer [1]. During surgical resection of a meningioma, CLI was used as a fast control for the resecting surgeon, confirming the presence of tumor-tissue in the excised mass by luminescence signal caused by $^{90}$Y-DOTATOC tumor-uptake, while the surrounding healthy brain tissue showed no radiotracer uptake. In addition to visual inspection during surgery, and resection-planning aided by the use of PET, MRI or CT, CLI adds information that cannot be provided by the other methods in such a setup – thus, CLI has a true added value not only for imaging-based antibody screenings, but also for resection-control directly flanking surgery.

### Author contributions

FCM and WMT designed the research. JS established tumor cell lines and animal models. RH provided antibodies. WMT and JS performed PET and MRI measurements. FCM and WMT performed CLI measurements, analyzed the data and conducted statistics. FCM and WMT wrote the manuscript. $^{64}$Cu was provided by WE and GR, antibody labeling was performed by AM. FCM, WMT, KN, RH and BJP discussed data and manuscript. All authors edited the manuscript.

## REFERENCES

1. Spinelli AE, Schiariti MP, Grana CM, Ferrari M, Cremonesi M and Boschi F. Cerenkov and radioluminescence imaging of brain tumor specimens during neurosurgery. J Biomed Opt. 2016; 21:050502-050502.

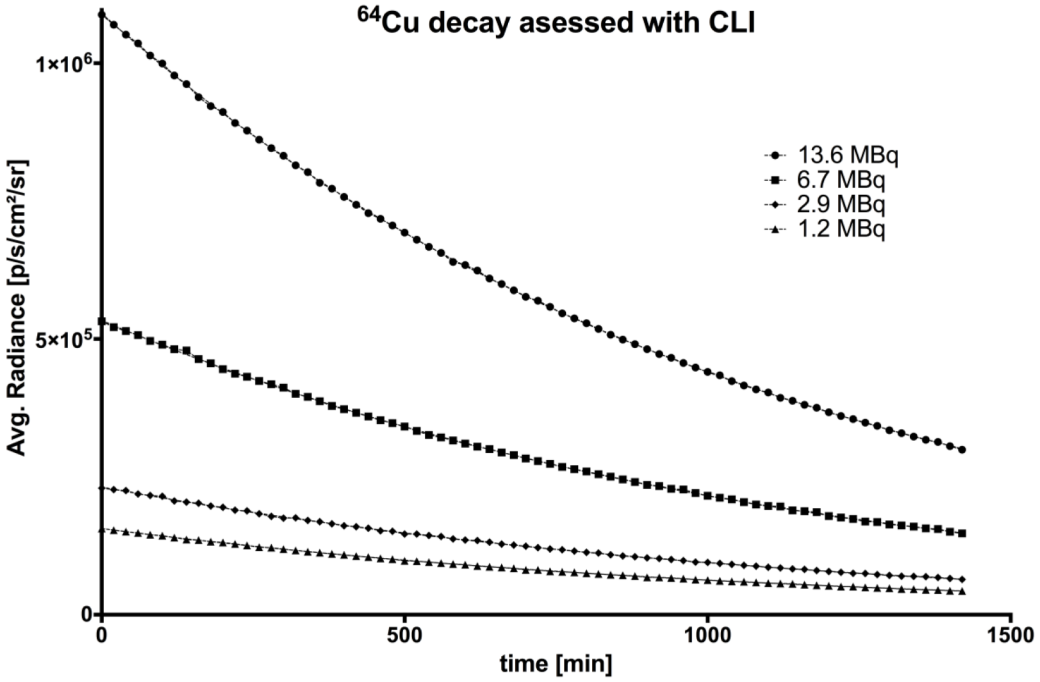


**Supplementary Figure S1: Phantom study assessing $^{64}$Cu-decay with CLI.** Phantoms filled with 100 µL PBS each, containing different activity concentrations of $^{64}CuCl_2$ (13.6 MBq, 6.7 MBq, 2.9 MBq and 1.2 MBq) were imaged over 24 h by means of CLI. Fitting of a mono-exponential function ($y = y_0 \cdot e^{-\tau_t t}$) well reflected the CLI-data and thus, mono-exponential radioactive decay ($R^2 = 0.99$ for all activity concentrations). Further, mono-exponential fitting revealed decay constants $\tau_t$ of 1.512*$10^{-5}$ 1/s, 1.511*$10^{-5}$ 1/s, 1.502*$10^{-5}$ 1/s and 1.516*$10^{-5}$ 1/s for the 13.6 MBq, 6.7 MBq, 2.9 MBq and 1.2 MBq activity concentrations, respectively.

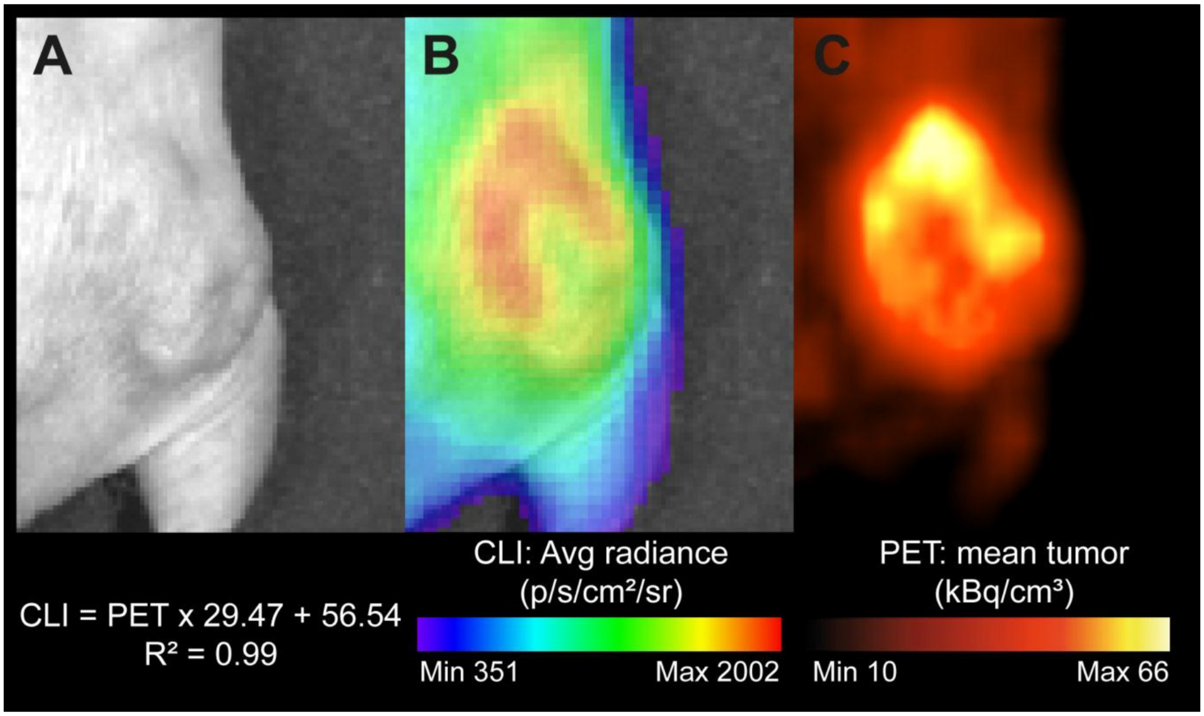


**Supplementary Figure S2: CLI and PET signal intensity normalized images 48 h after $^{64}$Cu-DOTA-ch14.18 injection.** To gauge the extent of signal variation with anatomy, the similarity and overall quality of CLI and PET images, CLI- and PET- signal intensity normalized images 48 h after $^{64}$Cu-DOTA-ch14.18 injection are shown (for the same LS-bearing SHO-mouse injected with $^{64}$Cu-DOTA-ch14.18 as displayed in Figure 2). Signal intensity was normalized using the linear correlation equation from Figure 3c. **A.** Photograph for CLI-overlay shown in **B. C.** Respective PET-MIP (image smoothing: 1 mm Gaussian).
